# Cost-Effectiveness Evaluation of a Novel Integrated Bite Case Management Program for the Control of Human Rabies, Haiti 2014–2015

**DOI:** 10.4269/ajtmh.16-0785

**Published:** 2017-06-07

**Authors:** Eduardo A. Undurraga, Martin I. Meltzer, Cuc H. Tran, Charisma Y. Atkins, Melissa D. Etheart, Max F. Millien, Paul Adrien, Ryan M. Wallace

**Affiliations:** 1 Health Economics and Modeling Unit, Division of Preparedness and Emerging Infections, National Center for Emerging and Zoonotic Infectious Diseases, Centers for Disease Control and Prevention, Atlanta, Georgia; 2 Poxvirus and Rabies Branch, Division of High Consequence Pathogens and Pathology, National Center for Emerging and Zoonotic Infectious Diseases, Centers for Disease Control and Prevention, Atlanta, Georgia; 3 Haiti Country Office, Division of Global Health Protection, Center for Global Health, Centers for Disease Control and Prevention, Port-au-Prince, Haiti; 4 Direction Production et Santé Animale, Protection Sanitaire, Ministère de l'Agriculture, des Ressources Naturelles et du Développement Rural, Port-au-Prince, Haiti; 5 Epidemiology, Laboratorie and Research, Ministère de la Santé Publique et de la Population, Port-au-Prince, Haiti

## Abstract

Haiti has the highest burden of rabies in the Western hemisphere, with 130 estimated annual deaths. We present the cost-effectiveness evaluation of an integrated bite case management program combining community bite investigations and passive animal rabies surveillance, using a governmental perspective. The Haiti Animal Rabies Surveillance Program (HARSP) was first implemented in three communes of the West Department, Haiti. Our evaluation encompassed all individuals exposed to rabies in the study area (*N* = 2,289) in 2014–2015. Costs (2014 U.S. dollars) included diagnostic laboratory development, training of surveillance officers, operational costs, and postexposure prophylaxis (PEP). We used estimated deaths averted and years of life gained (YLG) from prevented rabies as health outcomes. HARSP had higher overall costs (range: $39,568–$80,290) than the no-bite-case-management (NBCM) scenario ($15,988–$26,976), partly from an increased number of bite victims receiving PEP. But HARSP had better health outcomes than NBCM, with estimated 11 additional annual averted deaths in 2014 and nine in 2015, and 654 additional YLG in 2014 and 535 in 2015. Overall, HARSP was more cost-effective (US$ per death averted) than NBCM (2014, HARSP: $2,891–$4,735, NBCM: $5,980–$8,453; 2015, HARSP: $3,534–$7,171, NBCM: $7,298–$12,284). HARSP offers an effective human rabies prevention solution for countries transitioning from reactive to preventive strategies, such as comprehensive dog vaccination.

## Introduction

Rabies is a viral zoonosis that imposes a substantial burden in many developing countries, with approximately 59,000 annual deaths globally.^1^–^3^ Rabies transmission occurs in more than 150 countries and affects roughly half of the world's population. Human rabies is almost certainly fatal once clinical symptoms appear, but can be prevented if the bite victim is promptly administered postexposure prophylaxis (PEP).^4^ There are numerous rabies reservoir species globally; however, the vast majority of human rabies deaths are due to the virus variant enzootic in domesticated dog populations. Controlling dog rabies substantially reduces human exposure to the virus,^1^,^5^ which can be accomplished through periodical mass dog vaccination.^1^,^6^,^7^ But the costs of vaccination campaigns can be substantial.^8^ The Republic of Haiti has the highest human rabies burden in the Western Hemisphere,^9^,^10^ with an estimated 130 annual human deaths.^2^ As in other developing countries,^1^,^2^,^8^,^11^–^16^ many human and dog rabies cases are not recognized and are not reported to health authorities, thus limiting rabies awareness, funding, and prevention efforts.^10^ Dog bite victims often do not present to health-care facilities and PEP supplies may be limited.^2^,^17^,^18^ Budget constraints have resulted in limited passive rabies surveillance, inadequate laboratory capacity, few trained professionals, and NBCM (thus, all dog bites are treatedas suspected rabies exposures).^10^,^17^,^19^ Government-sponsored dog vaccination campaigns in Haiti have not been conducted to the frequency and intensity required for rabies elimination.^19^

In collaboration with the U.S. Centers for Disease Control and Prevention (CDC), the Haitian government initiated in 2013 the Haiti Animal Rabies Surveillance Program (HARSP), a form of integrated bite case management combining community bite investigations and passive animal rabies investigations to provide tailored rabies risk assessments for persons potentially exposed to the rabies virus.^10^ HARSP was established in three stages, beginning in 2011–2012 with the establishment of an animal rabies diagnostic facility. Stage 2 (2012–2013) involved the training of animal surveillance officers, including animal rabies surveillance, bite investigations, and use of equipment for safe and humane animal capture. The final stage was the implementation of the program. Investigations to locate biting dogs were triggered by a bite victim presenting for medical treatment in sentinel and nonsentinel hospitals or by reporting of suspect animals from the community. Offending animals were either euthanized or confined for observation, and potential bite victims were traced, advised to seek PEP, and referred to health-care facilities. In the first 2 years of operation, HARSP established a much higher dog rabies burden than previously recorded (∼18-fold increase in reporting of rabid animals), and averted numerous deaths from human rabies (∼3-fold increase in PEP of people with probable rabies exposure).^9^,^10^,^17^

While comprehensive dog vaccination is the ideal for rabies prevention and control, integrated bite case management programs may offer an efficient rabies-prevention and control solution for countries with high risk of rabies transmission and insufficient dog rabies vaccination coverage. Dog bite investigations allow removing rabid dogs from the community thus reducing potential exposures, and encouraging bite victims to seek appropriate medical care. By establishing reliable case definitions, bite victims of negative dogs can avoid unnecessary PEP. These programs also improve surveillance data, which can be used to assess disease burden, evaluate intervention programs and strategies, inform policy-makers, and focus on disease elimination.^8^

**Figure 1.**
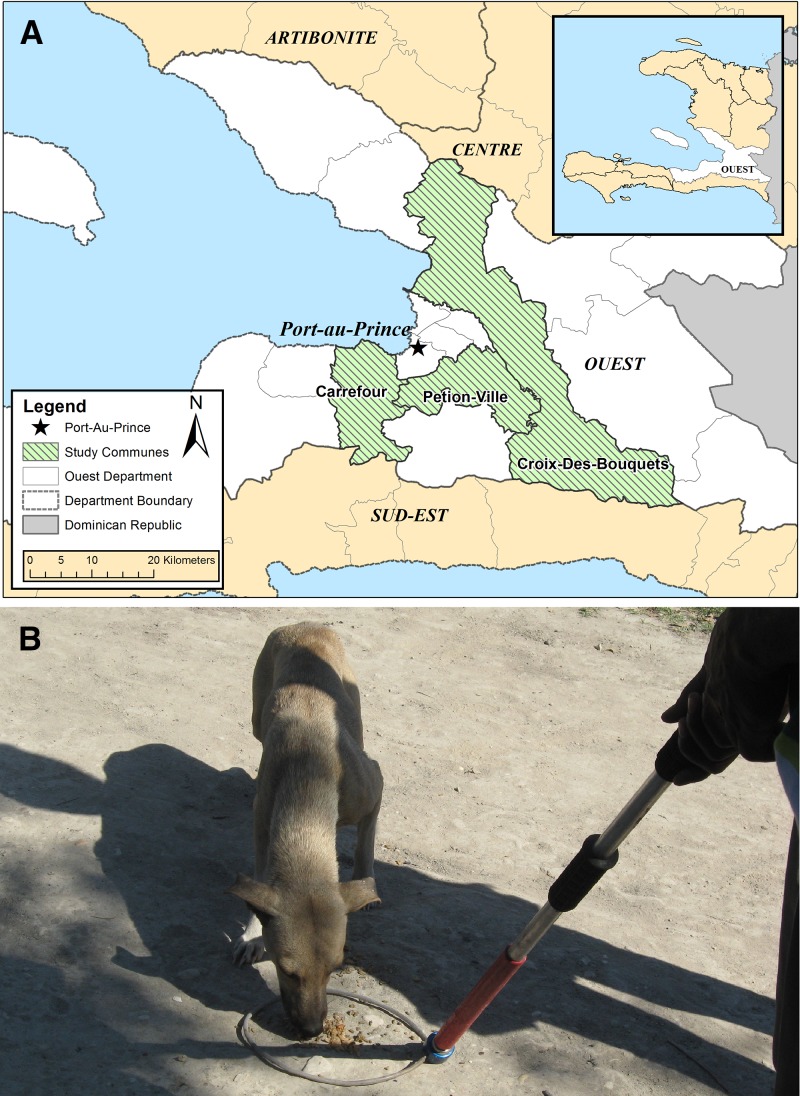
Location of the intervention and implementation of the rabies control program, Haiti, 2014–2015. (**A**) Map of the region where the Haiti Animal Rabies Surveillance Program was implemented. The program was initiated in three communes of the West Department (Ouest), Pétionville (population = 359,615; 89% urban), Carrefour (population = 487,980; 96% urban), and Croix-des-Bouquets (population = 238,222; 47% urban).^32^ (**B**) A rabies control officer uses food and a control pole to capture a free-roaming dog for a rabies assessment. HARSP had an active bite investigation component which helped identify an additional 40% bite victims.

Herein, we present the results from a cost-effectiveness analysis of the implementation of an integrated bite case management program, HARSP, in Haiti for the 2 years, 2014 and 2015, from the perspective of the government. We limit our analysis to the locations in which HARSP was first implemented: the West Department communes of Pétionville, Carrefour, and Croix-des-Bouquets (Figure 1

). These communities were chosen based on available infrastructure, such as roads and medical care facilities, and represent the range of socioeconomic conditions that prevail in Haiti. They are largely urban and probably have higher dog vaccination rates than most of the country due to recent annual dog vaccination campaigns.^10^ The results from this evaluation are intended to provide public and government entities affected by dog rabies with evidence to inform policy decisions about program replication.

## Materials and Methods

### Model.

HARSP implementation began in 2013 in Pétionville and was fully operational in all three communes by 2014. Using epidemiological and cost data from 2014 to 2015, we estimated the annual costs and effectiveness of HARSP from a governmental perspective. We developed a user-friendly spreadsheet (Supplemental File 1), where further details about calculations are provided. We estimated the costs and effectiveness of four alternative ways of implementing human rabies prevention programs (Table 1). The four scenarios assessed were 1) No bite case management (NBCM), that is, the medical treatment of dog bite victims that report to the health system with suspected rabies exposures (the situation in Haiti before the implementation of HARSP); 2) HARSP intervention, including animal rabies surveillance, bite investigations, and medical treatment of bite victims that report to the health system, including those potential victims advised to seek PEP during community bite investigations; 3) HARSP recommended (HARSPr), the hypothetical situation in which recommendations for rabies prevention and PEP treatment by HARSP are fully adhered; and 4) World Health Organization recommended (WHOr), the hypothetical situation in which all bite victims that seek medical treatment receive PEP according to the WHO recommendations.^8^ The latter two scenarios represent the potential for improvement of HARSP and NBCM, respectively, because they illustrate the results of rabies prevention and control if people strictly followed recommended guidelines. We considered the same self-directed bite victims' health-care-seeking behavior in the four scenarios; however, in the HARSP scenarios, we also added the proportion of bite victims that did not present to the health-care system initially but were encouraged to seek treatment as a result of dog bite investigations in their communities. We compared all four scenarios based on the number of rabies exposed persons (*n*_2014_ = 837; *n*_2015_ = 1,373), as assessed by HARSP during 2014 and 2015.

### Epidemiologic data.

Epidemiologic data included total human exposures, type of exposure,^8^ average number of PEP vaccines administered, and estimated rabies cases. The baseline rabies-exposure data were based on the results of HARSP animal rabies investigations. HARSP and HARSPr were the only scenarios in which information about categories of dog rabies exposure (confirmed, probable, suspected, negative) would be known because of the animal assessment component. Bite victims in NBCM and WHOr would have been treated as having suspected rabies exposures since no further investigation or classification of the offending animal would have been pursued (Table 2). However, not all bite victims who sought health care in the NBCM initiated PEP.

### Cost data.

Cost inputs included diagnostic laboratory development (start-up and maintenance), training of surveillance officers, operational costs (bite investigations, rabies surveillance, laboratory diagnosis), and also costs associated with rabies exposures and treatment of suspected rabies exposures. All costs were adjusted to 2014 dollars using gross domestic product implicit price deflators^21^ and program costs were annualized. Equipment for diagnostics included fluorescent microscope, incubator, freezer (solar), and fume hood (useful life: 5 years for all, except microscope: 10 years). For capital costs, we used the equivalent annual cost for the capital outlay considering that the resale value is zero. For the vehicles and most investments we considered a useful life of 5 years (details in Supplemental File 2, Appendix A). The costs of training in HARSP and HARSPr were prorated through 5 years (with no adjustment for inflation or discounting since the investment was on the first year).

We used previous studies to obtain the costs per dose of human rabies vaccine,^12^ unit costs of cold-chain (based on estimates of cold-chain costs for a measles vaccine in Haiti),^22^ and costs per outpatient visit based on WHO choice estimates for a public health facility in Haiti.^23^ Because we had no specific cost data for surveillance and diagnostics in Haiti prior to HARSP, we estimated the following values, based on the rabies diagnostic and surveillance activities, personnel, and equipment in place before the implementation of HARSP: 1) for the diagnostic facility, we considered a −20°C freezer, light microscope, 60% of the costs of equipment maintenance (compared with HARSP), $200 in reagents, 10% of HARSP costs for office supplies, same personnel costs, same office rental and utilities; 2) no costs of training personnel, and 3) 10% of HARSP costs in surveillance. Last, we estimated that the costs of the rabies surveillance and diagnostics for WHOr (scenario 4) were the same as those for NBCM (scenario 1); these scenarios only differed in rabies exposures treated and PEP compliance. None of these two scenarios (NBCM and WHOr) had a training component for active animal surveillance. The costs of the rabies surveillance, diagnostic, and training for HARSP and HARSPr were equivalent. Table 3 shows the costs of treating people with suspected rabies exposure, including PEP vaccines, rabies immunoglobulin (RIG), and outpatient visits, and basic cost input to estimate the costs of surveillance, diagnostics, and training (Supplemental File 2, Appendix A).

### Health and economic outcomes.

Per the four programmatic scenarios (NBCM, HARSP, HARSPr, WHOr), we estimated health outcomes as the total fatal human rabies infections and years of life lost (YLL) to premature death from rabies infection. Health outcomes were derived for each scenario from the estimated human rabies infections, calculated from the proportion of people bitten by a rabid dog (confirmed, probable, or suspected rabies), the probability that the bite victim was exposed to rabies, and the probability of acquiring rabies if exposed with no PEP. To estimate the number of fatal rabies infections, we combined the estimated human rabies infections with the probability that a bite victim sought medical care, including bite victims that were found in community bite investigations (Supplemental File 2, Appendix A). We assumed that patients who received PEP did not develop rabies, independent of overall compliance. For each scenario, we estimated cost outcomes as the total PEP vaccine doses administered and economic costs of the intervention. For patients with potential rabies exposure in the NBCM scenario, we considered the same compliance with PEP treatment as those who were exposed to suspected rabid dogs in HARSP, and the same criteria applied to RIG. We considered one outpatient visit per vaccine dose administration; the total estimated visits per patient were thus a function of treatment compliance. The acute phase of rabies typically lasts between 2 and 10 days; once symptoms appear the disease is almost always fatal.^24^ Few episodes of rabies, if any, are hospitalized.^2^ Furthermore, only a small proportion of rabies cases are identified through hospital-based reporting in Haiti.^19^ We thus excluded hospitalization costs. All outcomes were estimated based on the sample of patients who were reported to HARSP in 2014 and 2015 from any source. We summarized the cost-effectiveness of each scenario as the average cost per human rabies death averted and average cost per life year gained. Further details about calculations are shown in Supplemental File 2, Appendix A, and in Supplemental File 1.

We computed YLL by multiplying deaths at each age by the reference standard life expectancy at that age, based on the lowest age-specific death rates recorded and without age-weights or time preferences, for consistency with Global Burden of Disease studies.^25^ We used the age distribution of Haiti, assuming that Haiti had a similar rabies incidence by age as Tanzania.^11^ We also considered incidence by age data from Mexico^26^ and Ethiopia,^27^ but data were less complete and the resulting age distributions of rabies were similar to that of Tanzania.

### Uncertainty and sensitivity.

We conducted a multivariate sensitivity analysis addressing the main sources of data uncertainty: 1) share of PEP regimens paid by the government, 2) probability that bite victims would seek medical care, and 3) the probability that suspected rabid dogs were actually rabid. In 2013, Brazil donated about 20,000 Vero cell rabies vaccines for intramuscular administration^19^; however, a PEP program based on donations alone is unsustainable and the share of PEP costs currently subsidized by the government is uncertain. We thus present our results considering that the government pays for 1) no PEP costs (all is donated, including vaccine administration), 2) 50% of PEP costs (transitional status), and 3) 100% of PEP costs (self-sustaining status). We also show the variation of the main results using cost as a continuous variable.

Second, there is substantial variation in bite victims' health-care-seeking behavior. HARSP data show that 54% of bite victims sought medical care in the study area. However, a 2013 study in Pétionville estimated that 34% of bite victims sought care and only 31% initiated rabies vaccination.^28^ In contrast, 80% of rabies-exposed individuals sought medical care, but less than 65% initiated PEP in Tanzania.^15^ We used a range of 15–85% of bite victims presenting to a health-care facility to account for this uncertainty in the sensitivity analysis. We varied the share of PEP regimens paid by the government (0%, 50%, and 100%), the probability that bite victims would seek medical care (baseline HARSP data: 54%, estimated range: 15–85%), and the probability that suspected rabid dogs were actually rabid (baseline HARSP data: 6.3%, estimated range: 1–36%; upper bound was obtained from Hampson and others' estimate for Haiti).^2^

## Results

Table 4 shows the epidemiological parameters used in the evaluation, based on field data collected by HARSP. During 2014–2015, 54% of bite victims assessed by HARSP were reported from medical facilities. The remaining 46% of bite victims were found through active community bite investigations or direct reporting to HARSP from the victim. These individuals may have never sought medical care without HARSP. A retrospective study showed that 6% of bite victims did not seek medical care in 2015 despite HARSP advice to do so.^29^

The costs of animal surveillance by programmatic scenario are shown in Table 5. Dog investigations in 2014 (*N* = 778) resulted in 70 confirmed and 36 probable rabid dogs, and in 60 confirmed and 60 probable rabid dogs in 2015 (*N* = 1,657). These investigations resulted in 45 immediately euthanized dogs in 2014 and 47 in 2015 (Supplemental File 2, Appendix A). Dog investigations occasionally occurred beyond the limits of the three communes of the West Department.

Table 6 shows that HARSP substantially reduced the burden from rabies in the area of implementation. Among the 837 people reported to HARSP in 2014, we estimated 11 human deaths averted (14 under NBCM scenario to three deaths with HARSP compared), and 654 years of life gained (YLG) (832 YLL under NBCM to 178 with HARSP). For 2015, we estimated a total of nine human deaths averted among the 1,373 people assessed by HARSP (fatal rabies: 11 NBCM, two HARSP) and 535 YLG (YLL: 654 NBCM, 119 HARSP). Partly from a substantial increase in the number of identified bite victims and the associated PEP costs, independent from how much the government paid, the overall costs of the rabies prevention were always higher with HARSP compared with the scenario without HARSP (NBCM) (Table 6, 2014 [A] NBCM, vaccine doses: 290, costs: $15,988–$22,600; [B] HARSP, vaccine doses: 1,150, costs: $39,531–$64,750; 2015 [A] NBCM, vaccine doses: 477, costs: $16,025–$26,976; [B] HARSP, vaccine doses: 1,794, costs: $39,568–$80,290). However, HARSP had a considerably bigger impact in reducing human rabies, with approximately 11 deaths averted in 2014 and nine in 2015 compared with the NBCM scenario. Ultimately, HARSP was always more cost-effective than NBCM (2014: NBCM: $5,980–$8,453 per death averted, $101–$142 per YLG; HARSP: $2,891–$4,735 per death averted, $49–$80 per YLG; 2015: NBCM: $7,298–$12,284 per death averted, $123–$207 per YLG; HARSP: $3,534–$7,171 per death averted, $59–$121 per YLG).

**Figure 2.**
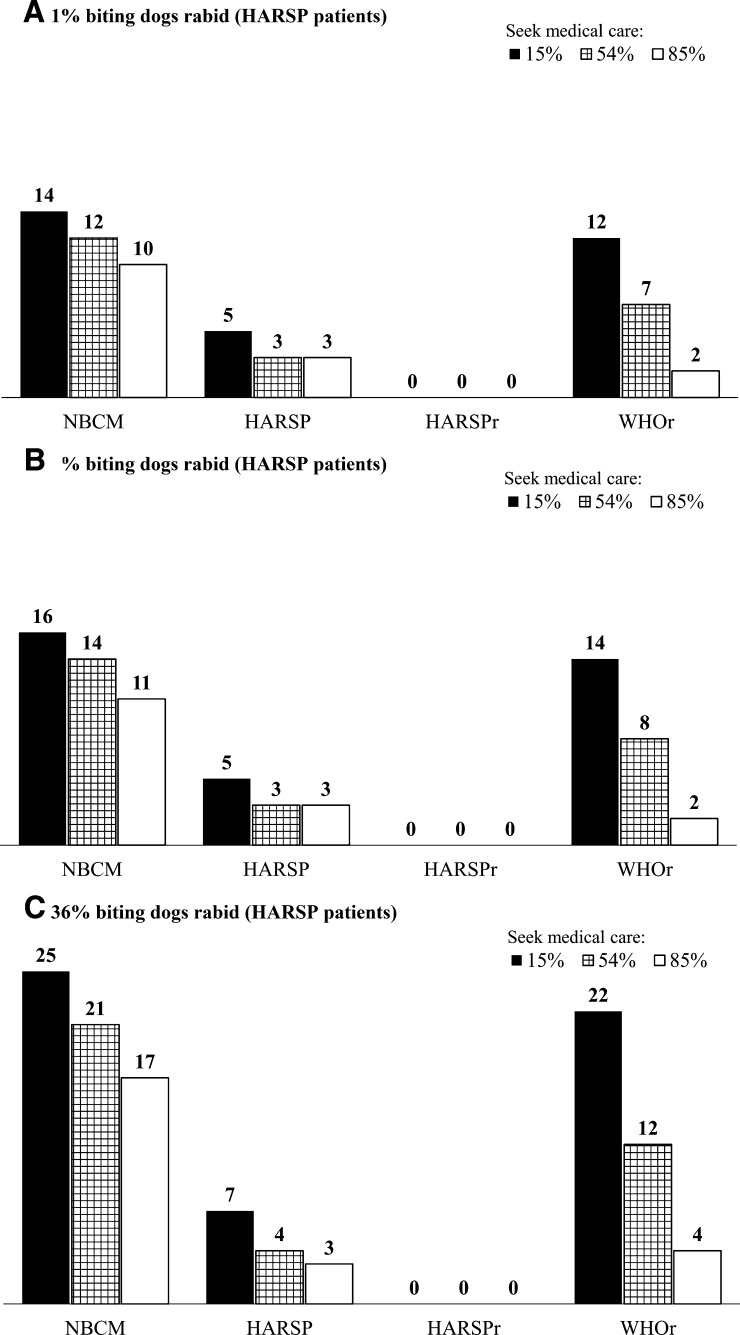
Two-way sensitivity analysis of the total fatal human rabies infections in the area of implementation of HARSP in 2014 by share of patients who seek medical care (%), and probability that a person bitten by a dog was exposed to rabies; (**A**) 1%, (**B**) 6.3% (estimate from HARSP), and (**C**) 36%(based on Hampson et al.'s estimates for Haiti). The figure shows results for 2014; results for 2015 are shown in the Supplemental File 2, Appendix B. **A**, **B**, and **C** show how the estimate for total fatal human rabies infections in the area of implementation of HARSP vary by the share and patients who seek medical care and the probability that the offending dogs among the HARSP population were actually rabid. HARSP = Haiti Animal Rabies Surveillance Program; HARSPr = Haiti Animal Rabies Surveillance Program (HARSP) recommendations for implementation of the program and rabies treatment; NBCM = no bite case management; WHO = World Health Organization; WHOr = World Health Organization recommendations for rabies treatment.

We found substantial variation in the expected number of human deaths from rabies when varying the share of patients who seek medical care (Figure 2

), particularly for NBCM and WHOr. Figure 2A–C shows how these results change by the probability that the offending dog (suspected rabid) was actually rabid. Because HARSP included dog bite investigations, changing the probability that the offending dog was rabid did not substantially affect the expected number of deaths from rabies for the HARSP scenarios.

Figure 3

**Figure 3.**
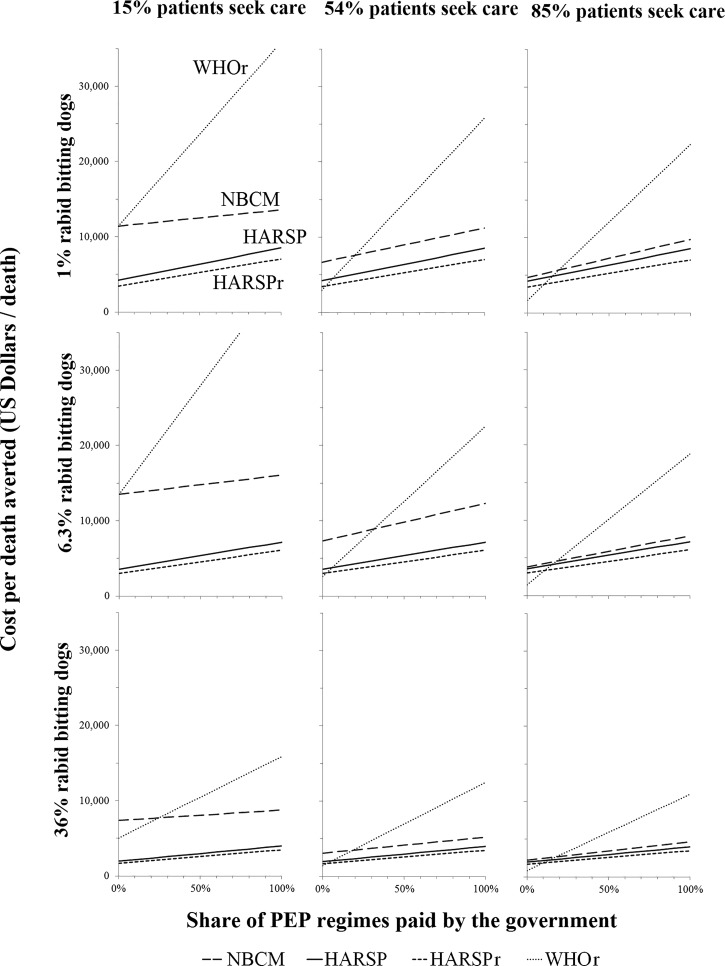
Multivariate sensitivity analysis: average cost per death averted (2014 US$/death) by share of patients who seek care (%), probability that a person bitten by a dog was exposed to rabies, and share of PEP costs paid by the government. The evaluation corresponds to year 2014. The estimated probabilities that a person bitten by a dog was exposed to rabies (1%, 6.3%, and 36%) were based on a plausible lower bound, data from HARSP project's dog investigations (average for 2014–2015), and estimates by Hampson and others' for Haiti,^2^ respectively. The share of patients who seek medical care was based on HARSP data (54%), and an illustrative range of 15–85%. HARSPr = Haiti Animal Rabies Surveillance Program (HARSP) recommendations for implementation of the program and rabies treatment; NBCM = no bite case management; PEP = postexposure prophylaxis; WHOr = World Health Organization recommendations for rabies treatment.

shows a multivariate sensitivity analysis of the estimated cost per death averted, varying the share of patients bitten by a suspected rabid dog who seek medical care and the share of PEP costs paid for by the government. The share of bite victims who seek medical care generated the greatest variation in results, especially for NBCM and WHOr. These two scenarios were also considerably affected by the share of suspected rabid dogs that actually have rabies. The results for WHOr also noticeably varied by the share of PEP costs paid by the government, because all exposures are treated as a suspected rabies exposure. Because HARSP included active community bite investigations, the program reached bite victims who did not seek care and offered PEP based on actual risk. Consequently, HARSP cost-effectiveness estimates were not greatly affected by changes in people's health-care-seeking behavior or in the probability that suspected rabid dogs had rabies.

## Discussion

HARSP substantively reduced the risk of human rabies death compared with the scenario without the program (NBCM) (additional annual averted deaths: 11 in 2014, nine in 2015; YLG: 654 in 2014 and 535 YLG in 2015). Implementing HARSP imposed a higher economic burden to the government of Haiti than the NBCM scenario (2014 NBCM: $15,988–$22,600; HARSP: $39,531–$64,750; 2015 NBCM: $16,025–$26,976; HARSP: $39,568–$80,290). The cost differences were largely explained by HARSP's active bite investigation component, which identified an additional 40% bite victims, some of which initiated PEP at an additional cost compared with non-HARSP scenarios. Costs also increased under HARSP scenarios due to improved PEP compliance. Dog bite investigations helped determine categories of dog rabies infection (i.e., confirmed, probable, suspected, and negative), which allowed for a more targeted use of PEP regimes for dog bite victims through communication of rabies risk based on case investigations. Under HARSP, there was higher compliance of full PEP regimes among confirmed and probable dog rabies exposures (78% and 37%, respectively) compared with suspected rabies exposures (33% compliance). This improvement in PEP compliance came at a slight cost increase of PEP per exposed patient who initiated treatment (NBCM: $37.6, HARSP: $38.8).

Rabies exposures constitute only a small proportion of dog bites in Haiti; therefore, there are potential savings in the administration of PEP regimes derived from delaying follow-up vaccination of bite victims based on a skilled animal assessor's evaluation of the offending dog. The government of Haiti has not formally adopted a policy to delay treatment based on a quarantine or testing outcome, and WHO recommendations do not include delaying PEP. While not considered in this economic evaluation, delaying follow-up PEP in relatively low-risk exposure scenarios where the animal is available for assessment by an experienced animal assessor (∼60% of potential exposures in HARSP) could reduce the costs of PEP while posing a negligible risk upon the exposed individual. The ideal of zero wastage of PEP on nonexposed individuals is unlikely, if not impossible, in dog rabies–endemic countries.

When considering both the costs and health outcomes, HARSP was more cost-effective than NBCM (2014: NBCM: $5,980–$8,453 per death averted, HARSP: $2,891–$4,735 per death averted; 2015: NBCM: $7,298–$12,284 per death averted, HARSP: $3,534–$7,171 per death averted), and the program has potential for improvement, as suggested by the HARSPr scenario results. From a societal perspective, that is, considering all costs and benefits irrespective of who pays and benefits from the intervention, the program would result in net benefits for the society as long as an additional year of life is valued at more than $121.

The effectiveness of HARSP may be even greater than estimated for at least three reasons: first, the rapid removal of rabid animals from the community may have reduced local dog-dog and dog-human rabies transmission, an effect that would be partly unaccounted for in this evaluation. In 2014 alone, 51 confirmed or probable rabid dogs were removed from these three communities. If we assumed that half of these dogs removed from the community bit just one additional person had HARSP investigators not been present to euthanize or confine the dog, we would expect about four additional human deaths when considering the parameters presented here. Routinely confining and observing biting dogs, independent of whether they are rabid or not, very likely resulted in fewer bite incidents and, as a consequence, a lower demand for PEP from suspected rabies exposures, although we did not consider this potential benefit in our cost estimates.

Second, the program may have decreased the risk of acquiring rabies beyond the three communes assessed, as dog investigations occasionally occurred beyond the three communes in the West Department. A previous evaluation of HARSP^10^ (2013–2015) had already established a much higher rabies burden in Haiti than the reported annual average of four dog and seven human rabies cases (2009–2012).^17^,^31^ Our results confirm that rabies burden is substantially underrecognized in Haiti. Consistently, a modeling study of rabies burden estimated 130 annual deaths in Haiti.^2^ If the rabies burden is proportional to the human population, we would expect ∼14 deaths in the three communes where HARSP was implemented (population^32^: 1,085,817), which is what we obtained in the NBCM scenario for 2014 (Table 6, [A]). Using the same criteria, we would expect an average of ∼48 annual deaths in the West Department under the NBCM (population^32^: 3,845,600). About 15 additional dog-mediated human deaths from rabies were averted by HARSP in the West Department, considering the overall effects of the program and assuming that half of the rabid dogs removed from the community would have bitten just one additional person. That represents a potential 31% decrease in the probability of dying from rabies in the West Department, simply by having a trained force of veterinary professionals available to respond to reports of suspected rabid animals (Supplemental File 2, Appendix B). Dividing by the total population in the West Department, the costs for this potential risk reduction were US$0.02 per person, that is, equivalent to ∼0.03% of the annual public and private health expenditures per person in Haiti (US$77.00, water and sanitation excluded).^33^

Third, the estimated improvement in cost-effectiveness of the HARSP scenario would be conservative if the observed increase in vaccination coverage in recent years decreased enzootic transmission. Haiti has supported dog mass vaccination programs consistently for the past 5 years. The results from a capture-recapture study (CDC, unpublished data) after the dog vaccination campaign of July 2014–April 2015 suggest that about 40% of the dog population in Haiti's West Department was vaccinated against rabies. While the impact of low-level vaccination coverage on dog rabies transmission is not well established, we observed a reduction in the proportion of observed dogs confirmed to be rabid in 2015 compared with 2014.

As with many developing countries, the Haitian government has limited resources that preclude the implementation of effective mass dog vaccination campaigns at this time.^17^ Reactive PEP-based solutions may reduce human rabies, as has happened, for example, in Vietnam.^34^ However, growing human and dog populations will likely result in ever-increasing costs, thus making this strategy nonsustainable.^14^ Health expenditures as a share of gross domestic product have increased in Haiti in the past decade from 4.4% in 2005 to 7.6% in 2014,^33^ but policy-makers face competing demands. Additional strategies of rabies prevention, not included in HARSP, may also help to optimize the use of limited resources. For example, child education programs and awareness campaigns may reduce rabies exposures and increase PEP use.^8^ Using intradermal regimes instead of intramuscular regimes of PEP administration could reduce the direct cost of vaccines by 60–80%.^1^,^18^ There is limited evidence that dog population management methods are effective at reducing the rates of dog rabies, although theoretically they could support vaccination campaigns; however, these programs can be very costly.^35^–^37^ Animal surveillance and control are usually in the realm of the ministry of agriculture, but a substantial share of the costs from rabies exposures fall on the ministry of health, which makes coordinating rabies prevention challenging. An intersectoral program, such as HARSP, can partially overcome these limitations by providing a more accurate estimate of rabies exposures in humans and prevalence in animals, and by encouraging both sectors to work together in a very practical way.

Our study has limitations. First, there is substantial uncertainty in the share of individuals potentially exposed to rabies who seek medical care. HARSP data suggested that 54% of potentially exposed individuals sought medical care in the study area, but this figure could be lower. Health-care-seeking behavior probably varies by rabies awareness, competing diseases, education, accessibility to health care, and travel costs, among other factors. Our sensitivity analysis suggests that the estimates for HARSP were not substantively affected by health-care-seeking behavior. Active community bite investigations resulted in a substantial increase in health-care-seeking behavior, which did not occur under a NBCM or WHO scenario. But the true number of bite victims who did not seek care remains unknown.

Second, we evaluated an intervention in three communes with closely monitored training and implementation of the diagnostic and surveillance components by international partners. The escalation of this intervention to a national level may not necessarily result in the same quality of operation, due to differences in accessibility, health-care quality, public infrastructure, funding, and others, among the different departments in Haiti.

Third, there were limited data about the burden of rabies in Haiti, which restricts the generalizability of our findings. For example, to estimate the YLL, we estimated the age distribution assuming that Haiti has similar incidence rates of rabies by age as Tanzania.^11^ The estimated age distribution from reported rabies deaths in Ethiopia^27^ was similar to Tanzania, and slightly skewed toward older age if compared with the distribution of reported dog-bite injuries in Mexico.^26^ Had we used the age distribution from Mexico, the estimated YLL would have been slightly higher in all scenarios, making HARSP look more favorable. Despite limited rabies surveillance data from Haiti, our estimates for rabies in the NBCM scenario are largely consistent with those of Hampson and others.^2^

Fourth, because we did not collect economic data prior to the implementation of HARSP, we used a cost structure for the animal rabies surveillance and diagnostics program under the WHOr and NBCM scenarios that was similar to that of HARSP, but we adjusted costs based on preexisting rabies diagnostic and surveillance activities, personnel, and equipment. Where necessary, we made conservative estimates of NBCM costs, to avoid overestimating the incremental cost-effectiveness ratios of HARSP.

Last, we only included costs per outpatient visits and assumed that no bite victim was hospitalized, as the evidence suggests that only a few episodes of rabies, if any, are hospitalized,^2^ probably because once symptoms appear the disease is almost always fatal.^24^ But we cannot confirm that this was always the case, due to limitations in reporting, surveillance, and laboratory capacity, and to the similarity of clinical symptoms of rabies with other diseases, such as cerebral malaria.^38^ Our results confirmed that rabies is underreported in Haiti, and our estimated deaths under the HARSP scenario seem reasonable and in agreement with previous estimates of rabies burden in the country.^2^,^10^

Overall, HARSP substantially reduced the expected risk of rabies transmission, resulting in more averted deaths and YLG, and was more cost-effective than the scenario without HARSP (NBCM). However, implementing and operating HARSP was more costly to the government than not having the program largely due to active community investigation components and the associated higher PEP costs. Additionally, HARSP helped to establish a better estimate of the disease burden of rabies in the three communes where the program operated, reduce the number of expected deaths from rabies exposures, and determine categories of dog rabies infections (i.e., confirmed, probable, suspected, and negative) for a more targeted use of PEP regimes. Improved surveillance data from HARSP can be used to track rabies disease burden, evaluate intervention programs and strategies, and focus on disease elimination. In sum, HARSP offers a cost-effective human rabies prevention solution for countries transitioning from reactive to preventive strategies (i.e., comprehensive dog vaccination). This evaluation is intended to provide policy-makers, donors, and the public affected by dog rabies with evidence to inform decisions about rabies prevention and program replication.

## Supplementary Material

Supplemental Files.

## Figures and Tables

**Table 1:** Evaluation of the HARSP and three comparison scenarios for rabies prevention under the same baseline conditions, Pétionville, Carrefour, and Croix-des-Bouquets communes, West Department, Haiti, 2014–2015

Scenario	Description
NBCM	Rabies prevention and control corresponds to the situation before HARSP was implemented, that is, passive surveillance, limited diagnostic capabilities, few trained health-care workers, and NBCM (i.e., treatment of dog bite victims that report to the health system as suspected rabies exposures).
HARSP	HARSP is a community-based animal rabies surveillance program with two components: active community bite investigation and passive animal rabies investigation. It includes updating laboratory and rabies surveillance capabilities.
HARSPr*	Activities related to rabies surveillance, rabies diagnostics, dog investigations, and treatment of suspected rabies exposures correspond to the best possible implementation of the HARSP program, strictly adhering to suggested guidelines and recommendations for the program.
WHOr*	Activities related to rabies surveillance, rabies diagnostics, dog investigations, and treatment of suspected rabies exposures correspond to the situation in Haiti NBCM, but all bite victims that seek medical treatment receive PEP according to WHO guidelines and recommendations for rabies treatment.

HARSP = Haiti Animal Rabies Surveillance Program; HARSPs = HARSP recommended; NBCM = no bite case management; PEP = postexposure prophylaxis; WHO = World Health Organization; WHOr = WHO recommended.

* Scenarios HARSPr and WHOr represent the potential for improvement, if guidelines and recommendations were strictly followed, of HARSP and NBCM (no HARSP).

**Table 2:** Baseline epidemiological data for the evaluation of the HARSP, Pétion-Ville, Carrefour, and Croix-des-Bouquets, West Department, Haiti, 2014–2015

Item	Units	Value		Source
		2014	2015	
Study population*	*N*	837	1,373	HARSP
Human exposures to rabies				HARSP
Confirmed	*N*	33	29	
Probable	*N*	59	39	
Suspected	*N*	166	177	
Negative	*N*	579	1,128	
Type of exposure (share)†				
Category I	%	0%	0%	Estimate
Category II	%	18%	16%	Wallace and others^10^
Category III (needs to add to 100%)	%	82%	84%	HARSP
Average PEP vaccines administered (HARSP)				HARSP
Confirmed	*N*	4.3	4.3	
Probable	*N*	2.4	2.4	
Suspected	*N*	3	3	
Negative	*N*	2.7	2.7	
Age distribution of rabies cases and exposures				Cleaveland and others^11^
0–4	%	9	9	
5–9	%	18	18	
10–14	%	18	18	
> 15	%	55	55	
Probability that suspected rabid dog had rabies infection	%	6.3	6.3	HARSP
Probability of acquiring rabies if exposed with no PEP‡	%	19	19	Shim and others^20^

HARSP = Haiti Animal Rabies Surveillance Program; PEP = postexposure prophylaxis; RIG = rabies immunoglobulin; WHO = World Health Organization. Most field data were collected by HARSP officers. Data correspond to 2014 and 2015; additional epidemiological data are shown in the Supplemental File 2, Appendix A.

* The study population included all persons who were potentially exposed to rabies and were in contact with HARSP or local health clinics.

† The types of contact were defined following WHO PEP recommendations, and are defined as follows^8^: Category I: touching or feeding animals, licks on the skin. Category II: nibbling of uncovered skin, minor scratches or abrasions without bleeding, licks on broken skin. Category III: single or multiple transdermal bites or scratches, contamination of mucous membrane with saliva from licks, exposure to bat bites or scratches. Category I requires no treatment, Category II requires immediate vaccination, and Category III requires immediate vaccination and RIG.

‡ The probability of acquiring rabies if exposed and not given PEP varies depending on several factors, including the type of exposure, anatomic site of the exposure, and severity. Another study in Tanzania,^16^ estimated that 14% of patients would have died had they not received PEP.

**Table 3:** Baseline cost data for the evaluation of the HARSP, in the communes of Pétionville, Carrefour, and Croix-des-Bouquets, West Department, Haiti, 2014–2015

Item	Units	Unit value	Sources
PEP			
Basic vaccine dose (including administration)*	$/dose	14.45	
Material costs (needles, swabs, etc.)	$/dose	0.12	Knobel and others^12^
Overhead costs per visit	$/visit	0.61	Knobel and others^12^
Tissue-culture vaccine	$/dose	12.20	Knobel and others^12^
Cold-chain	$/dose	0.05	Acharya and others^22^
Cost per outpatient visit†	$/outpatient	1.47	WHO-Choice^23^
Injections/doses per patient	*N*	5	Poxvirus and Rabies Branch
Vaccine doses per visit	*N*/visit	1	Recommendations
RIG‡	$	134.15	Knobel and others^12^
PEP treatment paid for by the government	(%)	(0, 50, 100)	Range used for estimates
Surveillance			
Vehicle (motorcycle) (annual)	$/vehicle	1,000	HARSP data
Maintenance (annual)	$/vehicle	100	HARSP data
Animal capture equipment	$/worker	800	HARSP data
Communications (mobile, radios, etc.)	$/worker	200	Estimated
Rabies prevention supplies (annual)	$/worker	500	HARSP data
Office rental§	$/year	4,000	Estimated
Utilities, supplies, etc.	$/year	1,200	HARSP data
Personnel (annual)	$/worker	3,300	HARSP data
Diagnostics			
Equipment	$/year	4,195	HARSP data
Equipment maintenance	$/year	500	Estimated
Rabies reagents	$/year	1,200	HARSP data
Supplies	$/year	5,000	HARSP data
Personnel (annual)	$/worker	6,006	HARSP data
Training∥			
Teacher (year)	$/training	1,487	HARSP data
Implementation (supplies, participants, etc.)	$/training	301	HARSP data

HARSP = Haiti Animal Rabies Surveillance Program; PEP = postexposure prophylaxis; RIG = rabies immunoglobulin; WHO = World Health Organization. Additional cost data and details on calculations are shown in the Supplemental File 2, Appendix A.

* Millien and others^19^ reported a stock of more than 15,000 does of human vaccine for PEP in stock (November 2014), from 20,000 Vero cell rabies vaccines for intramuscular administration donated by Brazil in 2013. Those authors also reported an annual use rate of about 8,000 human vaccine doses.

† WHO-Choice^23^ estimates: for a public facility. We used the average estimated costs for an outpatient visit of 1.47 (health center = $1.21, health center with beds = $1.50, primary-level hospital = $1.71, and secondary-level hospital = $1.78) and adjusted the cost to 2014 US$.

‡ The overhead and material costs from RIG were included in the basic PEP treatment of category III exposures.

§ If owned, we estimated the cost per square meter for an office in the same area. Office rental and utilities costs were considered separately for laboratories and rabies surveillance. If the office space was shared, we adjusted the value by multiplying it by the share of time dedicated to rabies.

∥ These costs represent the costs of training prorated in 5 years. See Supplemental File 2, Appendix A, for details.

**Table 4:** Epidemiological and health-care-seeking behavior data from comparison scenarios for patients with suspected rabies exposure who sought medical care or were identified through community bite investigations, Pétion-Ville, Carrefour, and Croix-des-Bouquets, West Department, Haiti, 2014–2015

Scenarios	Units	NBCM	HARSP	HARSPr	WHOr
Health-care-seeking behavior					
Share of patients who seek medical care*	%	54%	54%	54%	54%
Additional patients who seek medical care as a result of the bite investigation†	%	0%	40%	46%	0%
Share who did not seek care, despite HARSP advice			6%	0%	
Of patients who seek care, % that start PEP					
Confirmed	%	18%	100%	100%	100%
Probable	%	44%	68%	100%	100%
Suspected	%	41%	100%	100%	100%
Negative	%	39%	78%	0%	100%
PEP treatment					
PEP (recommended vaccine doses)	*N*	5	5	5	5
Of those who start PEP vaccines, % compliance					
Confirmed	%	33%	78%	100%	100%
Probable	%	33%	37%	100%	100%
Suspected	%	33%	33%	100%	100%
Negative	%	33%	33%	0%	100%
RIG	*N*	0.82	0.82	0.82	0.82
Share of category III exposures	%	N/A	N/A	82%	82%
Of those who get PEP vaccines, % receive RIG					
Confirmed	%	13%	11%	82%	82%
Probable	%	13%	0%	82%	82%
Suspected	%	13%	13%	82%	82%
Negative	%	13%	13%	0%	82%
Fatal human rabies infections‡	*N*	14	3	0	8
Confirmed	*N*	6	0	0	3
Probable	*N*	6	3	0	4
Suspected	*N*	2	0	0	1
Negative	*N*	0	0	0	0
Treatment setting					
Outpatient visits	*N*	5	5	5	5

HARSP = Haiti Animal Rabies Surveillance Program; HARSPr = HARSP recommendations for implementation of the program and rabies treatment; NBCM = no bite case management; PEP = postexposure prophylaxis; RIG = rabies immunoglobulin; WHO = World Health Organization; WHOr = WHO recommendations for rabies treatment.

* Percentage of patients, out of the total sample of patients who were reported to HARSP (by any means), that were reported to HARSP from a medical institution in Haiti.

† Share of patients who seek medical care as a result of active bite investigations following a bite report from a suspected rabid dog. A retrospective study of suspected rabies exposures in Pétionville, Haiti, in 2013 (Etheart and others, unpublished data) showed that 6% of suspected rabies exposures did not seek medical care despite HARSP advice. The NBCM scenario did not include active bite investigations, so the percentage is zero. WHO does not currently recommend active bite investigation in their guidelines.

‡ Fatal human infections were estimated based on people's medical care–seeking behavior and the probability that the bite victim was exposed to rabies. We assumed that any patient who received PEP treatment did not develop rabies, independent of compliance with PEP schedules.

**Table 5:** Summary of estimated annual costs of the dog surveillance program by evaluation scenario (2014 U.S. dollars), Pétion-Ville, Carrefour, and Croix-des-Bouquets, West Department, Haiti, 2014–2015

Animal rabies diagnostic facility	NBCM	HARSP
Animal rabies diagnostic facility	$5,184	$12,920
Capital costs	1,787	4,195
Operational costs	1,435	6,935
Personnel	1,962	1,790
Animal rabies surveillance program	$10,804	$24,823
Capital costs	44	1,572
Operational costs*	6,237	8,263
Personnel	4,488	14,988
Required trainings HARSP	$0	$1,788
Operational costs	0	301
Personnel	0	1,487
Total annual costs	$15,988	$39,531

HARSP = Haiti Animal Rabies Surveillance Program; NBCM = no bite case management. The full list of items considered for cost calculations of the surveillance program are shown in the Supplemental File 2, Appendix A. We used constant dollars (no inflation) and a discount rate of 3% for capital investments.^30^

* There were negligible differences in the operational costs of the HARSP program due to differences in the number of dogs that were euthanized in 2014 and 2015. HARSP euthanized 45 dogs in 2014 and 47 in 2015 (see Supplemental File 2, Appendix B). The number of dogs under observation varied substantially from 2014 (*N* = 453) to 2015 (*N* = 1,189), but dogs put in observation have no additional costs to the government.

**Table 6:** Main cost-effectiveness outcomes from the implementation of the HARSP initiated in Pétionville, Carrefour, and Croix-des-Bouquets communes, West Department, Haiti, 2014–2015

Program indicators (annual)	Units	NBCM	HARSP	HARSPr	WHOr
		[A]	[B]	[C]	[D]
Evaluation year 2014					
Effectiveness of the intervention					
Fatal human rabies infections	*N*	14	3	0	8
YLL due to premature death*	*N*	832	178	0	475
PEP vaccine doses administered	*N*	290	1,150	1,290	2,260
Costs of the intervention (including biologics)					
Government pays no PEP costs (all donated)	US$	15,988	39,531	39,531	15,988
Government subsidizes 50% of PEP costs (transition)	US$	19,294	52,140	60,477	52,682
Government pays 100% of PEP costs (self-sustaining)	US$	22,600	64,750	81,422	89,376
Cost-effectiveness indicators					
Average cost per human rabies death averted					
Government pays no PEP costs (all donated)	US$/death	5,980	2,891	2,371	1,843
Government subsidizes 50% of PEP costs (transition)	US$/death	7,216	3,813	3,627	6,074
Government pays 100% of PEP costs (self-sustaining)	US$/death	8,453	4,735	4,883	10,304
Average cost per LYG					
Government pays no PEP costs (all donated)	US$/LYG	101	49	40	31
Government subsidizes 50% of PEP costs (transition)	US$/LYG	121	64	61	102
Government pays 100% of PEP costs (self-sustaining)	US$/LYG	142	80	82	173
Evaluation year 2015					
Effectiveness of the intervention					
Fatal human rabies infections	*N*	11	2	0	7
YLL due to premature death	*N*	654	119	0	416
PEP vaccine doses administered	*N*	477	1,794	1,225	3,707
Costs of the intervention (including biologics)					
Government pays no PEP costs (all donated)	US$	16,025	39,568	39,568	16,025
Government subsidizes 50% of PEP costs (transition)	US$	21,501	59,929	59,988	77,822
Government pays 100% of PEP costs (self-sustaining)	US$	26,976	80,290	80,409	139,618
Cost-effectiveness indicators*					
Average cost per human rabies death averted					
Government pays no PEP costs (all donated)	US$/death	7,298	3,534	2,998	2,586
Government subsidizes 50% of PEP costs (transition)	US$/death	9,791	5,353	4,546	12,560
Government pays 100% of PEP costs (self-sustaining)	US$/death	12,284	7,171	6,093	22,534
Average cost per LYG					
Government pays no PEP costs (all donated)	US$/LYG	123	59	50	44
Government subsidizes 50% of PEP costs (transition)	US$/LYG	165	90	77	211
Government pays 100% of PEP costs (self-sustaining)	US$/LYG	207	121	103	379

HARSP = Haiti Animal Rabies Surveillance Program; LYG = life-year gained; NBCM = no bite case management; PEP = postexposure prophylaxis; WHOr = WHO recommendations for rabies treatment; YLL = years of life lost.

* A lower cost-effectiveness ratio indicates that the program achieves the same health outcome at a lower average cost.
