# Supplementary material for: Cost-Effectiveness Evaluation of a Novel Integrated Bite Case Management Program for the Control of Human Rabies, Haiti 2014–2015
Source: Am J Trop Med Hyg. 2017 Jun 7;96(6):1307–17. doi: 10.4269/ajtmh.16-0785 (PMC5462564; doi:10.4269/ajtmh.16-0785)
Supplement: Supplementary file 2 [file SD2_file2.pdf]

**Supplementary material**

# Cost-effectiveness evaluation of a novel integrated bite case management program for the control of human rabies, Haiti 2014 - 2015

Eduardo A. Undurraga, Martin I. Meltzer, Cuc H. Tran, Charisma Y. Atkins, Melissa D. Etheart, Max F. Millien, Paul Adrien, and Ryan M. Wallace

*American Journal of Tropical Medicine and Hygiene*

## Table of contents

1 Appendix A. Economic and epidemiological input for the program evaluation ..................... 2

1.1 Implementation of HARSP: costs of surveillance, diagnostics, and training .................. 2

1.2 Epidemiological inputs for the evaluation of HARSP .................................................... 6

1.3 Fatal rabies infections.......................................................................................................... 8

2 Appendix B. Other results from the program ........................................................................ 9

2.1 Cost and cost-effectiveness of the program ...................................................................... 9

2.2 Sensitivity analysis ........................................................................................................... 11

2.3 Estimates extrapolated to the West Department ............................................................ 12

3 Appendix C. Main assumptions used in the model ............................................................... 13

4 References for the supplementary material ........................................................................... 15

# 1 Appendix A. Economic and epidemiological input for the program evaluation

Appendix A shows the economic and epidemiological inputs used to evaluate the Haiti Animal Rabies Surveillance Program (HARSP).

## 1.1 Implementation of HARSP: costs of surveillance, diagnostics, and training

Table A1 shows the costs of capital and operation during the implementation of HARSP in Petionville, Carrefour, and Croix-des-Bouquets, Haiti. Table A2 shows the annual capital and operational costs of the animal rabies diagnostic facility costs, and Table A3 shows the annual costs of training the personnel involved in the implementation of the HARPS program. We used a 3% discount rate to estimate the costs of capital investments.

**Table A1.** Capital and operational costs of the animal rabies surveillance program, HARSP (annual) Pétion-Ville, Carrefour, and Croix-des-Bouquets, Haiti, 2014-2015*

| Item | Units | Unit value | Total units | Useful life (yr.) | Annual costs (US $)† |
|---|---|---|---|---|---|
| **Capital costs** | | | | | **$ 1,572** |
| Vehicles | N | | 4 | | |
| Cost per vehicle (motorcycle) | $/vehicle | 1,000 | | 5 | 873 |
| Animal capture equipment | $/worker | 800 | | 5 | 524 |
| Communications (mobile, radios, etc.) | $/worker | 200 | | 5 | 175 |
| **Operational costs** | | | | | **$ 8,263** |
| Rabies prevention supplies | $/worker | 500 | | | 1,500 |
| Dogs investigated and found alive‡ | N | | 531 | | |
| Dogs euthanized§ | N | | 45 | | |
| Sedation drug 1 | $/dog | 2.95 | 45 | | 133 |
| Sedation drug 2 | $/dog | 2.95 | 45 | | 133 |
| Euthanasia drugs | $/dog | 0.82 | 45 | | 37 |
| Disposal | | | | | |
| Incineration | $/dog | 20 | 45 | | 180 |
| Burying | $/dog | 10 | 45 | | 360 |
| Share of dogs buried | % | 80% | | | |
| Dogs under observation | N | | 444 | | |
| Additional costs per observation | $/dog | 0 | 444 | | 0 |
| Dogs quarantined | N | 9 | | | |
| Costs of home observation | $/dog | 0 | | | 0 |

| | | | | |
|---|---|---|---|---|
| Vehicles | N | | 4 | |
| Maintenance | $/vehicle | 50 | 4 | 200 |
| Gas, lubricants, etc. | $/vehicle | 50 | 4 | 200 |
| Insurance | $/vehicle | 0 | 4 | 0 |
| Office rental¶ | $ | 4,000 | | 4,000 |
| Utilities (electricity, gas, water, etc.) | $ | 1,000 | | 1,000 |
| Office items and supplies | $ | 200 | | 200 |
| Share of time dedicated to rabies | % | 100% | | |
| Communications (air time, maintenance, etc.) | $/worker | 30 | | 120 |
| Informatics | $ | 200 | | 200 |
| **Personnel** | | | | **$ 14,988** |
| Surveillance veterinary technicians | N | | 3 | |
| Share of time dedicated to rabies | % | 100% | | |
| Salary/wage# | $/worker | 3,000 | 3 | 9,000 |
| Fringe benefits | $/worker | 300 | 3 | 900 |
| Overtime payments | $/worker | 0 | 3 | 0 |
| Personal protective equipment** | $/worker | 200 | 3 | 600 |
| Program coordinator | N | | 1 | |
| Share of time dedicated to rabies | % | 100% | | |
| Salary/wage | $/worker | 4,080 | 1 | 4,080 |
| Fringe benefits | $/worker | 408 | 1 | 408 |
| Overtime payments | $/worker | 0 | 1 | 0 |

**Notes**: *We used constant dollars (no inflation) and a real discount rate.[1]
†These estimates show the equivalent annual cost for the capital outlay assuming that the resale value is zero. The "clinical" life of an equipment is usually less than its physical life, so were being conservative in our estimate.
‡The total costs of surveillance, including personnel, transport, diagnostic tests, and related costs were included as operational costs. We included the costs per dog euthanized, home observation of dogs that depend solely on the total number of dogs investigated (i.e., drugs for sedation, euthanization, and the costs of disposal).
§ For sedation, Xylazine: Ketamine = 2: 10. To produce complete anesthesia, 2 ml / 10 kg IM; for lethal injection (euthanasia), 2mEq/mL; dose: 2mmol/kg or 1mL/kg intracardiac.[2, 3]
¶If owned, estimate the cost per $m^2$ for an office in the same area. Office rental and utilities costs were considered separately for labs and rabies surveillance. If the office space was shared, the value was multiplied by the share of time dedicated to rabies.
#The cost per worker day was based on the monthly salary of a veterinary technician (Centre) of US$275. We considered that 10% of these costs correspond to fringe benefits.
**Annual costs of personal protective equipment and equipment for laboratory work, assuming that they wear out in a year.

**Table A2.** Capital and operational costs of the animal rabies diagnostic facility, HARSP (annual) in Pétion-Ville, Carrefour, and Croix-des-Bouquets, Haiti, 2014-2015*

| Item | Units | Unit value | Total units | Useful life (yr.) | Annual costs (US$)† |
|---|---|---|---|---|---|
| **Capital costs** | | | | | **$ 4,195** |
| Fluorescent microscope | $ | 22,000 | 1 | 10 | 2,579 |
| Incubator | $ | 1,000 | 1 | 5 | 218 |
| Freezer (solar) | $ | 1,400 | 1 | 5 | 306 |
| Fume hood | $ | 5,000 | 1 | 5 | 1,092 |
| **Operational costs** | | | | | **$ 6,935** |
| Equipment maintenance | $ | 500 | | | 500 |
| Insurance | $ | 0 | | | 0 |
| Rabies reagents | $ | 1,200 | | | 1,200 |
| Supplies for handling samples | $ | 5,000 | | | 5,000 |
| Office rental‡ | $ | 2,000 | | | 200 |
| Utilities (electricity, gas, water, etc.) | $ | 200 | | | 20 |
| Office supplies | $ | 150 | | | 15 |
| Share of time dedicated to rabies | % | 10% | | | |
| **Personnel** | | | | | **$ 1,790** |
| Laboratory technicians | N | | | | |
| Share of time dedicated to rabies | % | 10% | | | |
| Salary/wage | $/worker | 5,460 | 3 | | 1,575 |
| Fringe benefits | $/worker | 546 | 3 | | 158 |
| Overtime payments | $/worker | 0 | 3 | | 0 |
| Protective clothing and equipment$^{\chi}$ | $/worker | 200 | 3 | | 58 |

**Notes:** *We used constant dollars (no inflation) and use a real discount rate.[1]

†These estimates show the equivalent annual cost for the capital outlay assuming that the resale value is zero. The "clinical" life of an equipment is usually less than its physical life, so we were conservative in estimating the years of useful life.

‡ If owned, estimate the cost per $m^2$ for an office in the same area. Office rental and utilities costs were considered separately for labs and rabies surveillance. If the office space was shared, the value was multiplied by the share of time dedicated to rabies.

**Table A3.** Training cost for the implementation of the HARSP program in Pétion-Ville, Carrefour, and Croix-des-Bouquets, Haiti, 2014-2015

| Item | Units | Unit value | Total units | Total (US$) | Annual costs (US $) |
|---|---|---|---|---|---|
| **Operational costs** | | | | **$ 1,504** | **$ 301** |
| Classroom days | N | 5 | | 692 | 138 |
| Field days | N | 5 | | 692 | 138 |
| Number of participants | N | | 4 | | |
| Cost per day of each participant* | $/day | 35 | 4 | 138 | |
| Training supplies | $/day/worker | 25 | 4 | 100 | 20 |
| Training-related transport | $/day/worker | 5 | 4 | 20 | 4 |
| Space rental† | $/day | 0 | 4 | 0 | 0 |
| **Personnel** | | | | **$ 7,434** | **$1,487** |
| Teacher | N | | 1 | | |
| Days in training destination‡ | N | | 12 | | |
| Salary/wage§ | $/day | 308 | 12 | 3,692 | 738 |
| Travel expenses (per diem, hotel)¶ | $/day | 266 | 12 | 3,192 | 638 |
| Air-tickets# | $ | 550 | 1 | 550 | 110 |

**Notes:** The costs of training were prorated through the time frame of the project evaluation (with no adjustment for inflation or discounting since the investment was on the first year).
*The cost per day per participant was based on the salary/wage and fringe benefits of participants in the workshop; the figure shows a weighted average of the costs per worker involved in the animal surveillance program.
†We assumed that the classes took place in the facilities where the program was implemented, thereby the costs of renting a classroom were zero.
‡We estimated that a teacher would spend at least the equivalent to classroom days + field days + 2 days of settling in.
§Teacher's wage was estimated based on an annual salary of $80,000, assuming training from a CDC employee or equivalent, and 260 (=52*5) annual work days.
¶Per Diem allowances for Petionville, Haiti. Lodging: $155.00, meals and IE $110.00.[4]
#The price of air-tickets were estimated at market value.[5]

## 1.2 Epidemiological inputs for the evaluation of HARSP

Table A4 shows a summary of the epidemiological data from HARSP used in the effectiveness evaluation. The comparison scenarios for patients with suspected exposure to rabies in the intervention area of the program, including the situation in Haiti before the implementation of HARSP (no bite case management scenario, NBCM), the HARSP program, and the recommendations and guidelines for rabies treatment by HARSP (HARSPr) and the World Health Organization (WHOr) are shown in Table A5.

**Table A4.** Summary epidemiological data from HARSP initiated in Pétion-Ville, Carrefour, and Croix-des-Bouquets, Haiti, 2014-2015

| Variable | Units | Value | | Source |
|---|---|---|---|---|
| | | 2014 | 2015 | |
| **Study population** | N | 837 | 1,452 | HARSP data |
| **Human exposure to rabies*** | | | | |
| Confirmed | N | 33 | 31 | HARSP data |
| Probable | N | 59 | 63 | HARSP data |
| Suspected | N | 166 | 171 | HARSP data |
| Negative | N | 579 | 1187 | HARSP data |
| **Type of exposure (share)†** | | | | WHO[6] |
| Category I | % | 0% | 0% | Estimated |
| Category II | % | 18% | 16% | Wallace et al.[7] |
| Category III | % | 82% | 84% | HARSP data |
| **Potential dog-transmitted human rabies infect.** | | | | |
| Adjustment factor (Dog rabies/reported dog rabies)‡ | | 1 | 1 | |
| Adjusted bites (Reported dog rabies*adj. factor) | N | 258 | 245 | HARSP data |
| Confirmed | | 33 | 29 | HARSP data |
| Probable | | 59 | 39 | HARSP data |
| Suspected | | 166 | 177 | HARSP data |
| Negative (excluded from total) | | 579 | 1,128 | HARSP data |
| **Probability that person bitten was exposed to rabies§** | % | 6.3 | 6.3 | HARSP data |
| HARSP dog population* | | | | |
| Confirmed | % | 100 | 100 | |
| Probable | % | 75 | 75 | Assumption |

| | | | | |
|---|---|---|---|---|
| Suspected | % | 6.3 | 6.3 | HARSP data |
| Negative | % | 0 | 0 | |
| **Prob. of developing rabies if exposed with no PEP¶** | **%** | **19** | 19 | Shim et al.[8] |
| **Average PEP vaccines administered (HARSP)** | | | | |
| Confirmed | N | 4.3 | 4.3 | HARSP data |
| Probable | N | 2.4 | 2.4 | HARSP data |
| Suspected | N | 3.0 | 3.0 | HARSP data |
| Negative | N | 2.7 | 2.7 | HARSP data |
| **Human rabies infections in HARSP population** | **N** | **17** | **13** | |
| Confirmed | N | 6 | 6 | Calculated |
| Probable | N | 8 | 6 | Calculated |
| Suspected | N | 2 | 2 | Calculated |
| Negative | N | 0 | 0 | Calculated |
| **Dog-investigations HARSP** | | 778 | 1,657 | |
| Active surveillance | N | 40 | 35 | Wallace et al.[7] |
| Passive surveillance (located & non-located) | N | 738 | 1,622 | Wallace et al.[7] |
| Dogs investigated and found alive | N | 531 | 1,329 | Wallace et al.[7] |
| Dogs immediately euthanized | N | 45 | 47 | Wallace et al.[7] |
| Dogs placed under observation# | N | 453 | 1,189 | Wallace et al.[7] |
| Evaded capture | N | 33 | 93 | Wallace et al.[7] |
| Confirmed and probable dogs | N | 106 | 120 | Wallace et al.[7] |
| **Time frame for analysis** | Years | 5 | 5 | Definition |

**Notes:** HARSP denotes Haiti Animal Rabies Surveillance Program.
*****The categories of dog rabies infection (i.e., confirmed, probable, suspected, and negative) were used as a reference. HARSP and HARSPr were the only scenarios in which this information would be known; under WHOr and in the no bite case management scenario (NBCM), all patients would be treated as "suspected" rabid exposure.
†The types of contact are following WHO (2013)[6] PEP recommendations. Category I: touching or feeding animals, licks on the skin. Category II: nibbling of uncovered skin, minor scratches or abrasions without bleeding, licks on broken skin. Category III: single or multiple transdermal bites or scratches, contamination of mucous membrane with saliva from licks, exposure to bat bites or scratches. Category I requires no treatment, Category II requires immediate vaccination, and Category III requires immediate vaccination and RIG.
‡We are assuming that HARSP detected all potential human rabies cases in the study area.
§The probability that the offending dog had rabies was estimated based on empirical data, as described in Wallace et al.,[7] Figure 2. Probability = (confirmed + probable dogs) / total dog investigations. We used this probability for suspected dogs in the HARSP sample.
¶ Probability of developing rabies in the absence of PEP if bitten by a rabid dog.
#The number of dogs placed under observation in 2014 reflect 444 dogs that were observed for 14 days because they had bitten a human, and 9 dogs that were put in observation for 6 months because they were bitten a probably rabid dog. In 2015 there were 1,186 dogs under observation for 14 days, and 3 dogs under observation for 6 months.

## 1.3 Fatal rabies infections

Per the four programmatic scenarios (NBCM, HARSP, HARSPr, and WHOr), we estimated the total fatal human rabies infections as:

$$
\begin{aligned}
FR_{year} = \sum_{i=1\dots n} \sum_{j} P(ER_{ij}) \\
&\times \Big( P(MC_{ij}) \times \big( P(R_{ij}|PEP) \times P(PEP_{ij}) + P(R_{ij}|NoPEP) \times \big(1 - P(PEP_{ij})\big)\big) \\
&+ \big(1 - P(MC_{ij})\big) \\
&\times \Big( P(MC_{ij}|BI) \\
&\times \big( P(R_{ij}|PEP) \times P(PEP_{ij}) + P(R_{i,j}|NoPEP) \times \big(1 - P(PEP_{ij})\big)\big) \\
&+ \big(1 - P(MC_{i,j}|BI)\big) \times P(R_{i,j}|NoPEP)\Big)\Big)
\end{aligned}
$$

Where subscript *i* stands for a patient who was in contact with HARSP or local health clinics, i.e., is part of the study sample, *j* represents the type of exposure (i.e., confirmed, probably, suspected, negative).

$FR_{year}$ = fatal rabies infections (year = 2014, 2015)

$ER_{ij}$ = patient *i* with type of bite exposure *j* was exposed to rabies, i.e., $P(ER_{ij})$ is the probability that a patient *i* with bite exposure type *j* was exposed to rabies (confirmed = 100%, probable = 75%, suspected = 6.3%, negative = 0%)

$MC_{ij}$ = patient *i* with type of bite exposure *j* seeks medical care, i.e., $P(MC_{ij})$ is the probability that a patient *i* with bite exposure *j* seeks formal medical care

Rij = patient *i* with bite exposure *j* gets a rabies infection

PEP = Patient gets post-exposure prophylaxis

BI = bite investigation; the analyzed sample only includes bite victims who presented to a healthcare facility or were found through active community bite investigations.

Note that HARSP and HARSPr were the only scenarios in which information about categories of dog rabies exposure (confirmed, probable, suspected, negative) would be known because of the animal assessment component. Bite victims in NBCM and WHOr would have been treated as having suspected rabies exposures, since no further investigation of the offending animal would have been pursued. Further details about calculations and estimates used are shown in a spreadsheet attached as supplementary material, where assumptions can be easily modified.

## 2 Appendix B. Other results from the program

### 2.1 Cost and cost-effectiveness of the program

Here we show the results from the evaluation of the program based on the share of the costs of PEP regimes that is paid for by the government (0%, 50%, and 100%). Figure B1 shows that the total costs of the program (2014 US dollars, thousands) vary substantially based on the share of PEP costs that the government pays. Figure B2 shows the cost-effectiveness of the program as total costs per death averted and Figure B3 as the total costs per year of life gained. Independent on how much the government pays, the estimated net monetary value of the HARSP program was always higher than in the scenario with no bite case management (NBCM) in Haiti. However, the cost-effectiveness ratios (cost per death averted and cost per life year gained) were always more favorable for HARSP.

**Figure B1.** Economic costs (2014 US dollars, thousands) of HARSP and alternative interventions, assuming that the government pays for 0%, 50%, and 100% of PEP costs

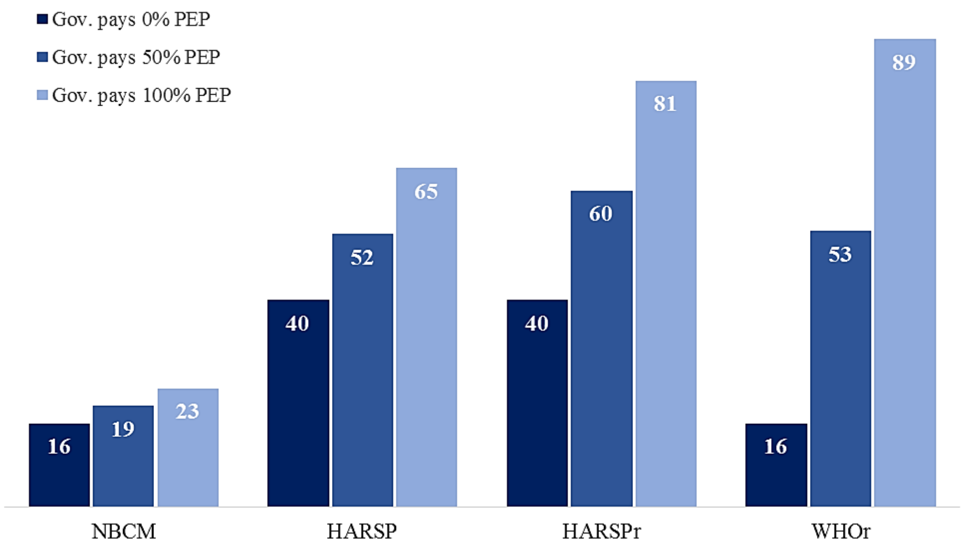

**Figure B2.** Average cost (2014 US dollars) per death averted from HARSP and alternative intervention scenarios, assuming that the government pays for 0%, 50%, and 100% of PEP costs

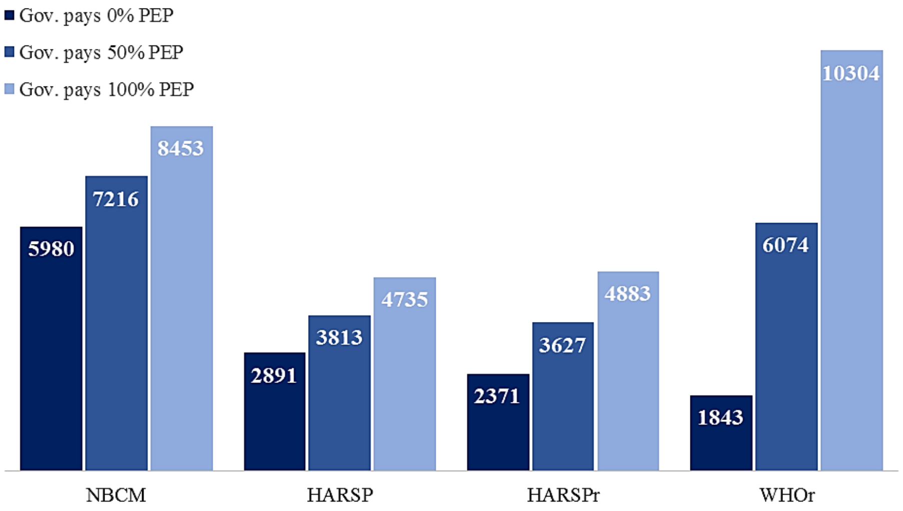


**Figure B3.** Average cost (2014 US dollars) per year of life gained from HARSP and alternative intervention scenarios, assuming that the government pays for 0%, 50%, and 100% of PEP costs

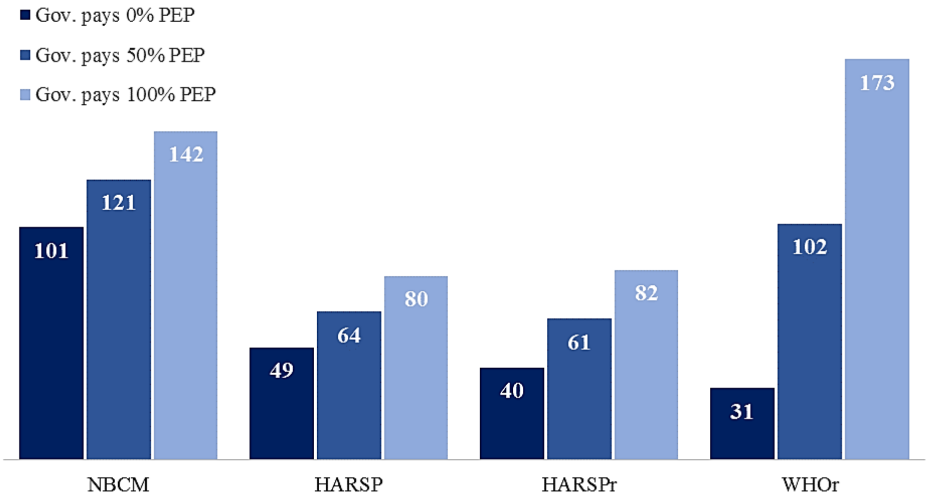


**Notes:** The figures shows the estimates for 2014. *WHOr denotes World Health Organization recommendations and guidelines for rabies treatment. HARSPr denotes Haiti Animal Rabies Surveillance Program (HARSP) recommendations and guidelines for implementation of the program and rabies treatment. ¶The age distribution to estimate years of life lost was based on the age distribution of Haiti, assuming that Haiti has the same incidence rates of rabies by age as Tanzania.[9]

## 2.2 Sensitivity analysis

**Figure B4.** Two-way sensitivity analysis of the total fatal human rabies infections in the area of implementation of HARSP in 2015 by share of patients who seek medical care (%), and probability that a person bitten by a dog was exposed to rabies, A: 1%, B: 6.3% (estimate from HARSP), and C: 36%.†

**A: 1% biting dogs among HARSP patients were rabid**

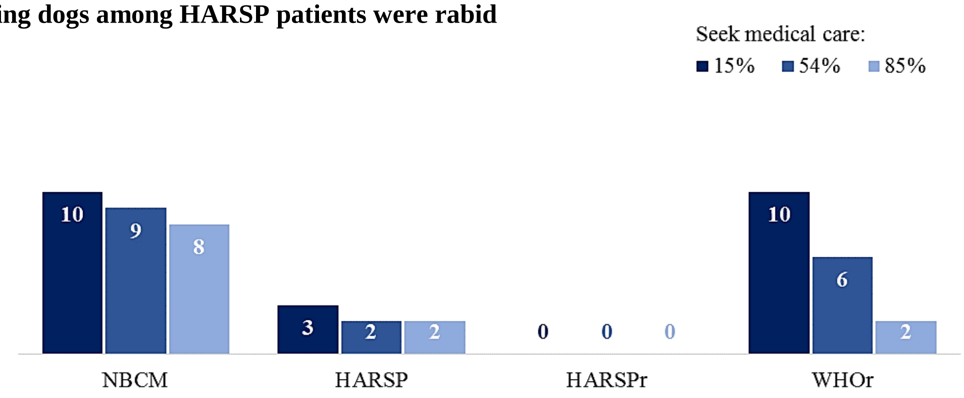


**B: 6.3% biting dogs among HARSP patients were rabid**

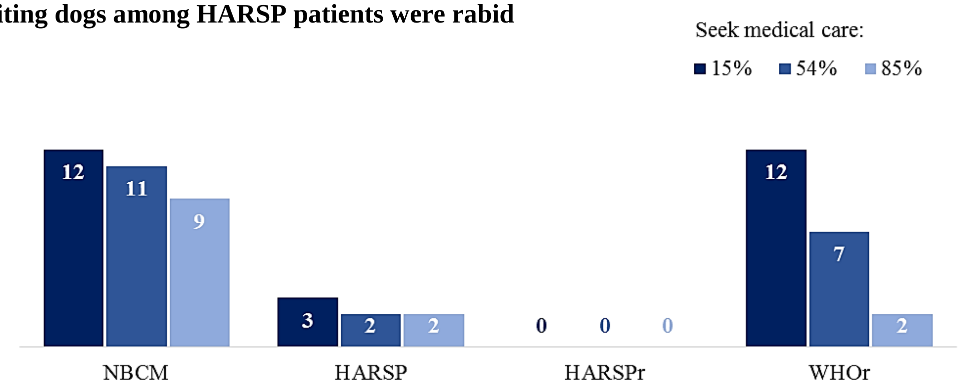


**C: 36% biting dogs among HARSP population were rabid†**

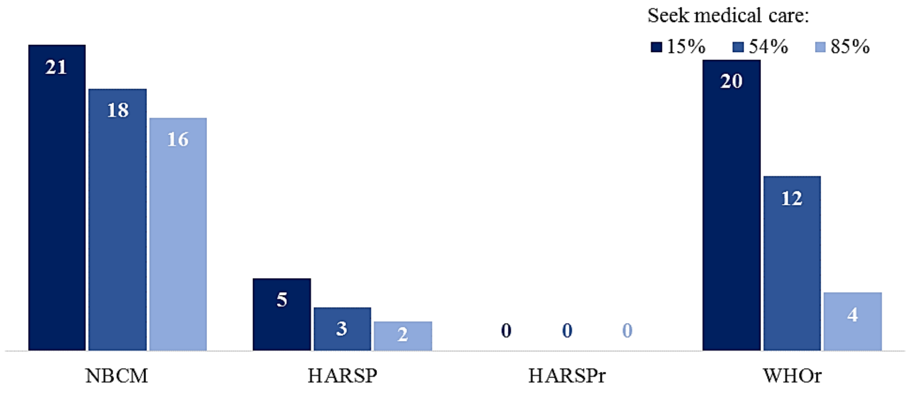


**Notes:** The figure shows results for 2015, results for 2014 are shown in the main manuscript. Figure 2A, 2B, and 2C show how the estimate for total fatal human rabies infections in the area of implementation of HARSP vary with the probability that the offending dogs among the HARSP population were actually rabid, A: 1%, B: 6.3% (estimate from dog investigations), and C: 36%. † 36% was based on Hampson et al.'s estimates for Haiti.[10]

## 2.3 Estimates extrapolated to the West Department

Combining literature and modeling, a study of the burden of rabies estimated 130 annual deaths from rabies in Haiti.[10] Assuming that the disease burden of rabies is proportional to the human population, we would have expected 14 deaths in the three communes where HARSP was implemented in 2014 (population:[11] 1,085,817), which is what we obtained in the NBCM scenario. Using the same criteria, we would expect an average of 48 annual deaths in the complete West Department (population:[11] 3,845,600). Dog investigations occasionally occurred beyond the three communes in the West Department. In 2014 (n=778) dog investigations resulted in 26 confirmed (n=24) or probable (n=2) rabid dogs euthanized and 25 dogs placed under observation (13 confirmed, 12 probable) in the West Department. To illustrate the relative impact of HARSP on rabies, if we assumed that half of these dogs would have bitten one additional person who would have received treatment as in the NBCM scenario, without HARSP we would have seen approximately 15 additional deaths from rabies in the West Department. Put another way, the implementation of HARSP, including the removal of suspect rabid dogs, resulted in a ~31% decrease in the overall probability of dying from rabies (Figure B4). Dividing by the total population in the West Department, the costs for this reduction in the probability of dying from rabies were US$0.02 per person. For context, the annual health expenditures per person in Haiti are about US$77.00,[12] including public and private expenditures (water and sanitation infrastructure not included).

**Figure B4.** Estimated fatal human rabies infections in the West Department, Haiti. Comparison of Haiti Animal Rabies Surveillance Program and alternative intervention scenarios (2014)

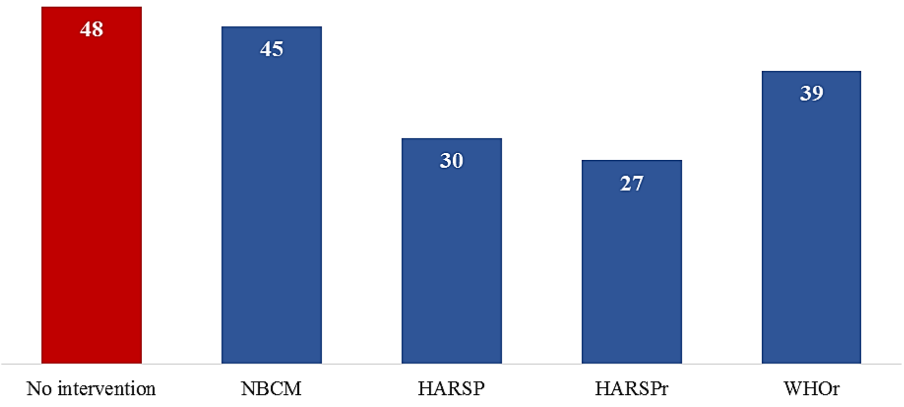


**Notes:** The figure shows results for 2014. * WHOr denotes World Health Organization recommendations and guidelines for rabies treatment. HARSPr denotes Haiti Animal Rabies Surveillance Program (HARSP) recommendations and guidelines for implementation of the program and rabies treatment. Deaths in the West Department derived from Hampson et al.[10] estimate of 130 annual deaths from rabies in Haiti, assuming a disease burden proportional to population size (West Department population:[11] 3,845,600; population in Pétion-Ville, Carrefour, and Croix-des-Bouquets:[11] 1,085,817).

## 3 Appendix C. Main assumptions used in the model

The following list shows a summary of the assumptions we used in our cost-effectiveness evaluation of Haiti Animal Rabies Surveillance Program (HARSP) from the perspective of the government of Haiti.

1. The same share of patients sought medical healthcare in the four scenarios analyzed.
2. The same number of post-exposure prophylaxis (PEP) vaccines doses (5) were recommended in all scenarios analyzed.
3. Patients with potential rabies exposure in Haiti had the same compliance with PEP treatment as those who were exposed to suspected rabid dogs in HARSP. The same applied to RIG.
4. Patients who received PEP vaccines did not develop rabies, independent of the patient's compliance with the PEP treatment. The probability that a patient developed rabies did not vary in the current model based on whether the patient got RIG or did not.
5. We considered that HARSP detected all potential human rabies cases in the study area.
6. The categories of dog rabies infection (i.e., confirmed, probable, suspected, and negative) were used only as a reference. HARSP and HARSP recommended were the only scenarios in which information about rabies exposure would have been known due to active bite investigation; under WHO recommendations and in the no bite case management scenario (NBCM), all patients would have been treated as "suspected" rabid exposures.
7. The total potential exposures to rabies only included the population that participated in the HARSP program, and the distribution of confirmed, probable, suspected, and negative exposures was the same for all scenarios analyzed.
8. If > 95% of reported potential exposures to rabies virus were bites, we estimate that all exposure were bites (category III by WHO standards). In our sample, 82% of bites were in category III in 2014 and 84% in 2015.
9. The incidence of human rabies infections by age in Tanzania[9] was equivalent to that of the study area in Haiti. We also considered other data (Mexico[13] and Ethiopia[14]), but the data were less complete and the resulting age distribution was similar.
10. Years of life lost from rabies were considered for the individuals' lifetime and not only for the 5 years of the project intervention.

11. We estimated life expectancy based on the lowest age-specific death rates recorded across countries in 2010, following Global Burden of Disease 2010 method.[15]
12. We used constant dollars (no inflation) and a discount rate of 3% for capital investments.[1]
13. We only included the costs of outpatient visits; we did not consider hospitalization. The evidence suggests that only a few episodes of rabies, if any, are hospitalized.[10]
14. We used one outpatient visit per PEP vaccine dose.
15. The costs of training were prorated through the time frame (5 years) of the project evaluation (with no adjustment for inflation or discounting since the investment was on the first year).
16. Costs of rabies surveillance and diagnostic in NBCM correspond to the situation in Haiti without the HARSP program. We estimated the following values, based on the rabies diagnostic and surveillance activities, personnel, and equipment in place before the implementation of HARSP: i) for the diagnostic facility, we considered a -20C freezer, light microscope, 60% of the costs of equipment maintenance (compared with HARSP), $200 in reagents, 10% of HARSP costs for office supplies, same personnel costs, same office rental and utilities; ii) no costs of training personnel, and iii) 10% of HARSP costs in surveillance.
17. We used the same costs for rabies surveillance and diagnostics in the Haiti NBCM scenario and WHO.

## 4 References for the supplementary material


1. Drummond MF, Sculpher MJ, Torrance GW, O'Brien BJ, Stoddart GL, 2005. Methods for the economic evaluation of health care programmes. Oxford, UK: Oxford University Press.

2. Kvsupply, 2016. Dog supplies, canine medications. Available at: http://products.kvsupply.com. Accessed 5 January, 2015.

3. Shopmedvet, 2016. Medical and veterinary supplies. Available at: http://www.shopmedvet.com/product/ Accessed 5 January, 2016.

4. FedTravel, 2015. Per Diem Rates. Available at: http://www.fedtravel.com/travel-tools/per-diem-rates.html. Accessed 28 December, 2015.

5. Kayak, 2015. Flights. Round-trip, ATL-PAP (prices vary constantly). Available at: www.kayak.com/flights Accessed 30 December, 2015.

6. World Health Organization, 2013. WHO Expert Consultation on Rabies. Available at: http://apps.who.int/iris/bitstream/10665/85346/1/9789240690943_eng.pdf. Accessed 30 March, 2016.

7. Wallace RM, Reses H, Franka R, Dilius P, Fenelon N, Orciari L, Etheart M, Destine A, Crowdis K, Blanton JD, Francisco C, Ludder F, Vilas VDR, Haim J, Millien M, 2015. Establishment of a canine rabies burden in Haiti through the implementation of a novel surveillance program. *PLoS Negl Trop Dis 9*: e0004245.

8. Shim E, Hampson K, Cleaveland S, Galvani AP, 2009. Evaluating the cost-effectiveness of rabies post-exposure prophylaxis: a case study in Tanzania. *Vaccine 27*: 7167-7172.

9. Cleaveland S, Fevre EM, Kaare M, Coleman PG, 2002. Estimating human rabies mortality in the United Republic of Tanzania from dog bite injuries. *Bull WHO 80*: 304-310.

10. Hampson K, Coudeville L, Lembo T, Sambo M, Kieffer A, Attlan M, Barrat J, Blanton JD, Briggs DJ, Cleaveland S, 2015. Estimating the Global Burden of Endemic Canine Rabies. *PLoS Negl Trop Dis 9*: e0003709.

11. Institute Haitien de Statistique et D'Informatique (IHSI), 2012. Population totale, population de 18 ans et plus menages et densites estimes en 2012. Available at: http://www.ihsi.ht/pdf/projection/DOC_POPTLE18_MENEST2012.pdf. Accessed 22 December, 2015.

12. World Bank, 2016. Data. Health expenditure per capita (current US$). Available at: http://data.worldbank.org/indicator/SH.XPD.PCAP Accessed 18 July, 2016.

13. Eng T, Fishbein D, Talamante H, Hall D, Chavez G, Dobbins J, Muro F, Bustos J, De Los Angeles Ricardy M, Munguia A, 1993. Urban epizootic of rabies in Mexico: epidemiology and impact of animal bite injuries. *Bull WHO 71*: 615.

14. Yimer E, Newayeselassie B, Teferra G, Mekonnen Y, Bogale Y, Zewde B, Beyene M, Bekele A, 2002. Situation of rabies in Ethiopia: a retrospective study 1990-2000. *Ethiop J Health Dev 16*: 105-112.

15. Murray CJ, Ezzati M, Flaxman AD, Lim S, Lozano R, Michaud C, Naghavi M, Salomon JA, Shibuya K, Vos T, 2012. GBD 2010: design, definitions, and metrics. *Lancet 380*: 2063-2066.
